# Retraction: Resistance to DDT and Pyrethroids and Increased kdr Mutation Frequency in *An*. *gambiae* after the Implementation of Permethrin-Treated Nets in Senegal

**DOI:** 10.1371/journal.pone.0156195

**Published:** 2016-05-19

**Authors:** Mamadou O. Ndiath, Seynabou Sougoufara, Abdoulaye Gaye, Catherine Mazenot, Lassana Konate, Oumar Faye, Cheikh Sokhna, Jean-Francois Trape

The authors of this article have requested retraction on the basis of concerns about the experimental work and the integrity of the reported data.

 An institutional investigation was conducted; the enquiry could not verify that the experiments underlying the results reported in the article had taken place. In light of the findings from the enquiry, the direction of the Institut de Recherche pour le Développement support the retraction of the article.

 In light of the above concerns, the authors retract this publication.
